# Virus-like particle – mediated delivery of the RIG-I agonist M8 induces a type I interferon response and protects cells against viral infection

**DOI:** 10.3389/fcimb.2022.1079926

**Published:** 2022-12-14

**Authors:** Enrico Palermo, Magdalini Alexandridi, Daniele Di Carlo, Michela Muscolini, John Hiscott

**Affiliations:** Pasteur Laboratories, Istituto Pasteur Italia - Fondazione Cenci Bolognetti, Rome, Italy

**Keywords:** virus like particle (VLP), RIG-I, antiviral immune response, innate immunity, type-I IFN, viral infection, SARS-CoV-2

## Abstract

Virus-Like Particles (VLPs) are nanostructures that share conformation and self-assembly properties with viruses, but lack a viral genome and therefore the infectious capacity. In this study, we produced VLPs by co-expression of VSV glycoprotein (VSV-G) and HIV structural proteins (Gag, Pol) that incorporated a strong sequence-optimized 5’ppp-RNA RIG-I agonist, termed M8. Treatment of target cells with VLPs-M8 generated an antiviral state that conferred resistance against multiple viruses. Interestingly, treatment with VLPs-M8 also elicited a therapeutic effect by inhibiting ongoing viral replication in previously infected cells. Finally, the expression of SARS-CoV-2 Spike glycoprotein on the VLP surface retargeted VLPs to ACE2 expressing cells, thus selectively blocking viral infection in permissive cells. These results highlight the potential of VLPs-M8 as a therapeutic and prophylactic vaccine platform. Overall, these observations indicate that the modification of VLP surface glycoproteins and the incorporation of nucleic acids or therapeutic drugs, will permit modulation of particle tropism, direct specific innate and adaptive immune responses in target tissues, and boost immunogenicity while minimizing off-target effects.

## Introduction

Since their discovery, Virus-Like Particles (VLPs) have gained interest as a potential therapeutic vehicle against infectious diseases or cancer (Mohsen and Bachmann, 2022; Tornesello et al., 2022). Several characteristics make VLPs an attractive alternative to conventional therapeutic approaches. Their structural similarity to native viruses provides self-assembly ability and natural tropism, whereas the absence of viral genome improves their safety by making them non-infectious (Noad and Roy, 2003; Smith et al., 2013). VLPs represent a next-generation vaccine platform, as they possess a stronger immunogenicity compared to traditional vaccines (Murata et al., 2003; Noad and Roy, 2003; Roldao et al., 2010; Safaeian et al., 2013; Guo et al., 2019): indeed, with a size ranging from 20 to 200 nm, VLPs are captured by antigen-presenting cells (APCs) and easily drained to lymph nodes (Bachmann and Jennings, 2010; Win et al., 2011; Mohsen et al., 2018). In addition, the expression of a high number of viral molecules organized in repetitive patterns on their surface, elicit a strong B and T cells adaptive immune response (Deml et al., 2005; Ross et al., 2009; Zabel et al., 2014). Different VLPs–based vaccines are commercially available, including those against HBV, HPV, HEV and Malaria (Mohsen and Bachmann, 2022). Recent advances in bioengineering have demonstrated the possibility to optimize VLPs immunogenicity by modifying surface protein expression and by encapsulating a variety of molecules such as peptides, proteins or nucleic acids (Banskota et al., 2022), thereby making VLPs a tissue- and cell- specific platform for drug delivery (Rohovie et al., 2017).

RIG-I is a key immune sensor in the innate antiviral response, and upon binding of 5’ di- and triphosphate short double-stranded RNA (dsRNA), triggers a signaling cascade with consequent activation of mitochondrial antiviral protein (MAVS), tank-binding kinase 1 (TBK1) and IκB kinase complex (IKK). These events result in the activation of interferon regulatory factors (IRF3 and IRF7) and NF-κB, with subsequent induction of antiviral and proinflammatory responses (Zevini et al., 2017; Rehwinkel and Gack, 2020). In addition, RIG-I signaling drives type I interferon (IFN)-dependent activation of dendritic cells (DCs), increasing surface marker expression and enhancing antigen processing (Castiello et al., 2019; Zevini et al., 2022). Once activated, DCs engage CD4^+^ T cells promoting a cell-mediated immune response as well as B cells maturation and antibodies production. (Gutjahr et al., 2016; Zevini et al., 2017).

We previously demonstrated that sequence modifications altering the length and structure of the original short 5’ppp-RNA derived from Vesicular Stomatitis Virus (VSV) (WT-5’ppp-RNA) generated a variety of short 5’ppp-RNAs that possess the ability to activate RIG-I, stimulating the antiviral and proinflammatory response to different degrees. Among them, M8 elicited a stronger antiviral response both *in vitro* and *in vivo*, inhibiting viral infections in primary human DCs and prolonging survival and reducing viral loads in mice challenged with influenza and chikungunya viruses (Beljanski et al., 2015; Chiang et al., 2015). In addition, M8 possesses anti-tumor activity by inducing immunogenic cell death in human cancer cells (Castiello et al., 2019).

Recently, inclusion of the STING agonist 2’-3’ cGAMP in SARS-CoV-2 Spike-enveloped VLPs enhanced the titers of SARS-CoV-2 neutralizing antibodies in mice (Chauveau et al., 2021), indicating that the delivery of molecular cargo activating the type I IFN signaling represents a potent immunogenic adjuvant during vaccination.

In this study, we used a mammalian cell line system to produce VLPs with incorporated M8 and evaluated the ability of these particles to target and release M8 into different types of cells *in vitro*, assessing the consequent induction of type I IFN response. VLPs-M8 efficiently entered A549, Huh-7 and Calu-3 cells, activating a strong antiviral response that inhibited VSV, DENV, hCoV-229E and VSV-CoV-2 Spike pseudotyped virus replication. Furthermore, VLPs-M8 displayed a strong therapeutic potential by blocking VSV replication after infection onset. In addition, we demonstrated that the expression of different surface glycoproteins, including the Spike protein of SARS-CoV-2, modified VLPs tropism to a specific target. These results highlight the efficiency of this delivery platform in terms of cellular targeting and activation of the antiviral response by combining VLPs and M8. Furthermore, the possibility to modify the VLPs surface by expressing different viral envelope proteins will allow the targeting of specific cell populations for drug delivery.

## Results

### M8 inclusion into enveloped VLPs

To generate a delivery platform with the ability to target specific cells and to induce an antiviral response, we produced virus-like particles (VLPs) by using the well described HIV-1 system (Arevalo et al., 2016; Martins et al., 2022) enveloped with VSV-G glycoprotein and incorporating a 5’ppp-RNA, termed M8, that triggers a type I IFN antiviral program upon binding to the RIG-I cytosolic sensor. As a negative control we used VLPs carrying CIAP-M8, in which the 5’ triphosphate group was removed from M8 by the Calf Intestinal Alkaline Phosphatase.

**Figure 1:**
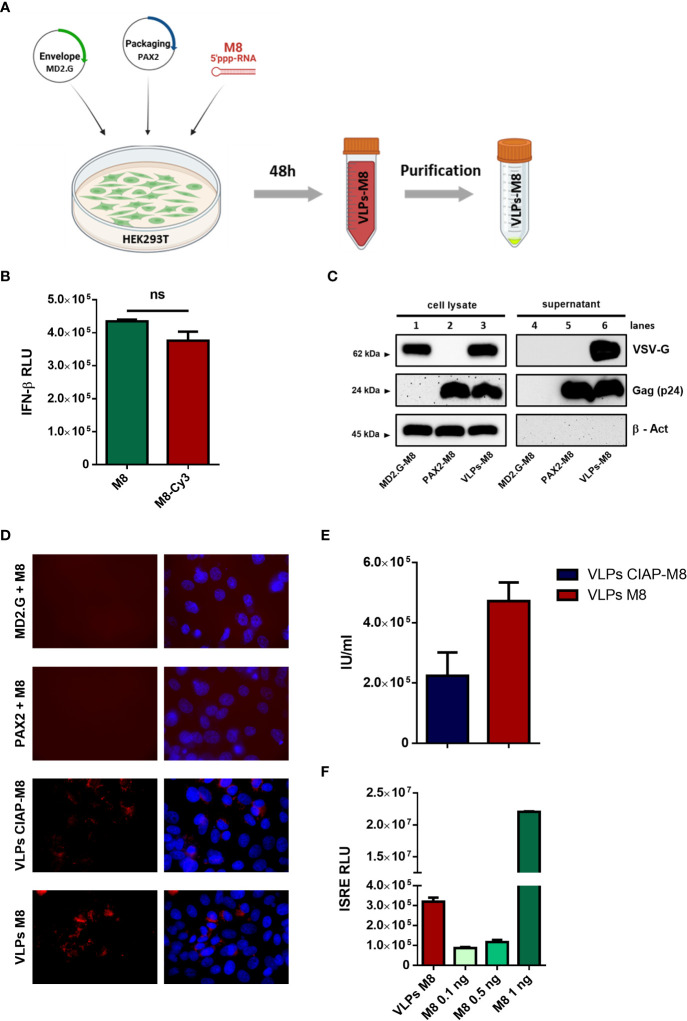
Generation and characterization of VLPs-M8 **(A)** Schematic representation of VLPs generation. HEK293T cells were seeded in a T-75 flask and after 24h were transfected with plasmids encoding for envelope and packaging, together with M8 or the negative control CIAP-M8. 48h after transfection supernatants were collected then purified and concentrated by ultracentrifugation. The figure was created with BioRender.com. **(B)** Comparison between M8 and M8-Cy3 induction of IFN response. M8 and M8-Cy3 were transfected in HEK293T reporter cell line expressing firefly luciferase under IFN-β promoter (IFN- β luc). Luciferase activity was measured 24h after transfection and indicates the induction of IFN-β promoter. (ns: not significant) **(C)** Immunoblot analysis representative of VSV-G and Gag-p24 protein expression in HEK293T whole cell extract and supernatant containing VLPs. Protein expression was normalized on β-Actin. **(D)** Representative images of M8-Cy3 and CIAP-M8-Cy3 localization. Analysis of VERO E6 cells seeded in a 24-well plate and treated for 48h with incomplete or complete VLPs, images were obtained using a Olympus fluorescence microscope (100x magnification) and analyzed by ImageJ. **(E)** Calculation of VLPs titer. VLPs-CIAP-M8 and VLPs-M8, both expressing Cyanine-3, were added to VERO E6 cells and the percentage of Cy-3 positive cells was measured after 48h by flow cytometry. VLPs titer was then calculated as described in Materials and Methods. **(F)** IFN signaling activity. Supernatants of A549 cells treated with VLPs-M8 (20µg) or M8 at the indicated concentrations, were collected after 48h of treatment and tested on HEK293T reporter cell line expressing firefly luciferase under ISRE promoter (ISRE-luc). Luciferase activity was measured 24h after treatment and indicates the induction of ISRE promoter.

VLPs were produced by co-transfecting HEK293T cells with M8 together with HIV-1 gag-pol and VSV envelope glycoprotein (VSV-G) plasmids, expressing packaging and envelope proteins, respectively (**Figure 1A**). After its intracellular assembly and incorporation of M8, the viral core is released by the cell and, during the budding process, acquires part of the cellular membrane including VSV-G protein expressed on its surface (Cervera et al., 2019).

To track the incorporation of M8 into VLPs, the 5’ppp-RNA was labeled with the fluorophore Cyanine-3 (M8-Cy3) using a commercial kit (Label IT, Mirus Bio). To verify whether the labeling of M8 could alter its ability to bind RIG-I and stimulate IFN production, an IFN-β promoter reporter assay was used by transfecting HEK293T cells with equal amounts of M8 and M8-Cy3 alongside a plasmid encoding for luciferase under the control of IFN-β promoter (IFN-β-luc). No significant differences were detected in the levels of IFN-β promoter activation, upon stimulation with M8 or M8-Cy3 (**Figure 1B**), thus demonstrating that the labeling of M8 did not impair its functionality. We then evaluated whether the expression of both packaging and envelope plasmids were necessary for VLPs formation, release and entry into target cells. HEK293T cells were transfected with packaging plasmid only, envelope plasmid only, or both. M8-Cy3 was present in each experimental condition and was used to monitor efficiency of VLPs entry and release of cargo into target cells. Western blot analysis of VLPs purified from supernatant revealed that the expression of packaging molecules was required for particle release (**Figure 1C** – lines 5&6) while the presence of VSV-G envelope was necessary for VLPs entry into VERO E6 cells (**Figure 1D**). To quantify the amount of VLPs incorporating M8 (VLPs-M8) or its inactive form (VLPs-CIAP-M8), VERO E6 cells were infected with VLPs-M8-Cy3 or VLPs-CIAP-M8-Cy3 and the VLPs titer was measured by flow cytometry 48h after treatment (**Figure 1E**). In addition, to quantify the stimulation of IFN signaling generated by VLPs-released M8, we used a reporter cell line expressing luciferase under the control of ISRE promoter (**Figure 1F**). HEK293T-ISRE-luc were treated with supernatant collected from A549 transduced for 48h with VLPs-M8 or increasing doses of directly transfected M8: analysis of luciferase expression showed that the induction of type I IFN signaling obtained with 2.5*10^3^ infection units (IU) of VLPs-M8 was comparable to that elicited by M8 at a concentration of ≈0.5 ng/mL (**Figure 1F**). Overall, these results demonstrate that the presence of both packaging and envelope proteins were necessary for M8 incorporation into VLPs and its delivery into cells; also, M8 released into target cells efficiently stimulated a type I IFN response.

### VLPs-M8 activate antiviral response and block VSV infection in A549 cells

**Figure 2:**
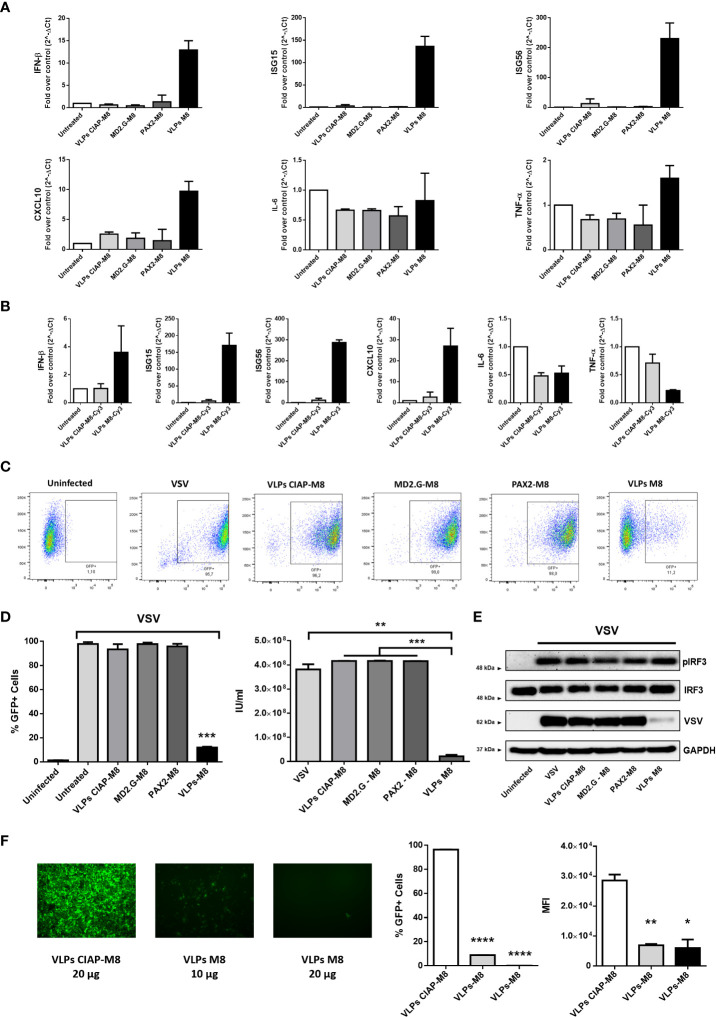
Antiviral potential of VLPs-M8 **(A)** Gene expression levels of IFN-β, ISG15, ISG56, CXCL10, IL-6 and TNF-α in A549 were quantified by qPCR after 48h of indicated treatments (20µg). Results are normalized to the β-Actin gene and expressed as fold of increase relative to control (Untreated), set as 1. **(B)** Gene expression levels of IFN-β, ISG15, ISG56, CXCL10, IL-6 and TNF-α in A549 were quantified by qPCR after 48h of treatment with 2.5*10^3^ IU of VLPs-M8-Cy3 or VLPs-CIAP-M8-Cy3, or left untreated. Results are normalized to the β-Actin gene and expressed as fold of increase relative to control (Untreated), set as 1. **(C)** Flow cytometry analysis representative of GFP expression in cells treated as indicated for 48h and then infected for 24h with VSV-GFP. **(D)** Analysis of VSV infection and replication in cells treated with VLPs. Left, histogram indicating the percentage of GFP positive A549 cells; right, viral titer calculated on VERO E6 cells treated with supernatant of A549 cells (left panel). **(E)** Immunoblot analysis representative of IRF3 activation and VSV-GFP protein expression in A549 whole cell extract. Cells were treated as indicated for 48h and infected with VSV-GFP (MOI 0.1) for 24h; protein expression was normalized on GAPDH. **(F)** Therapeutic potential of VLPs-M8. Left panels, images representative of A549 cells infected with VSV-GFP (MOI 0.001) and treated 1h post-infection with VLPs-CIAP-M8 or VLPs-M8 at the indicated concentrations, images were obtained using a Olympus fluorescence microscope (10x magnification) and analyzed by ImageJ. Central and right panels, flow cytometry analysis of GFP expression at 24hpi in A549 cells treated with VLPs post-infection; central panel indicates the percentage of GFP positive cells, right panel indicates the Mean Fluorescence Intensity. Data represent mean ± SEM from 3 independent experiments. Statistical significance was defined as follows: *p < 0.05, **p < 0.01, ***p < 0.001, and ****p < 0.0001.

To assess whether VLPs-M8 blocked viral replication, lung adenocarcinoma A549 cells were treated with VLPs and infected with VSV, a RNA virus that triggers a RIG-I – mediated host response. qPCR analysis of cells treated for 48h with VLPs-M8, VLPs-CIAP-M8 or incomplete VLPs (particles expressing envelope or packaging only) indicated that VLPs-M8 induced the expression of IFN-β and ISGs mRNA, while neither VLPs-CIAP-M8 nor incomplete VLPs stimulated IFN or ISGs gene transcription (**Figure 2A**). Interestingly, VLPs-M8 did not induce a strong proinflammatory response, suggesting that its activity promotes a robust antiviral response without triggering excessive, potentially detrimental, inflammation. We further confirmed the activation of type-I IFN response by treating A549 cells with VLPs-CIAP-M8-Cy3 and VLPs-M8-Cy3. Results demonstrated that although both VLPs were able to infect cells at the same extent (data not shown), the induction of interferon response was detected only in presence of VLPs incorporating M8-Cy3 but not with CIAP-M8-Cy3 (**Figure 2B**), and the levels were similar to that observed with VLPs-M8. Using an engineered VSV expressing GFP as a marker of viral replication (VSV-GFP), we demonstrated the ability of VLPs-M8 to protect A549 cells from infection. Indeed, flow cytometry analysis performed 24h post-infection (hpi) showed a marked decrease in the GFP_+_ cell population (**Figure 2C**), that correlated with a >80% reduction in infected cells (**Figure 2D**, left panel) and lower viral titer (**Figure 2D**, right panel), compared to infection alone or cells treated with VLPs-CIAP-M8 or incomplete VLPs. To further confirm these observations, we performed the analysis of protein expression which showed that activation of the RIG-I signaling occurs during VSV infection even in absence of VLPs-M8, as indicated by the phosphorylation of IRF3, that is related to a productive viral replication, and represents a physiological cellular response mechanism against the virus. However, in presence of VLPs-M8 this induction correlated with the block of viral replication, as evidenced by the reduced expression of viral products, demonstrating that VLPs-M8 elicited a strong IRF3-dependent antiviral response that limited infection progression (**Figure 2E**). These results indicate that VLPs-M8 can effectively target A549 cells and trigger an antiviral response that blocks VSV infection.

In addition, the activation of type I IFNs signaling resulted in a potent induction of antiviral genes expression more than a proinflammatory response, suggesting that M8 limited viral replication without exacerbating inflammation. Interestingly, treatment of A549 cells with VLPs-M8 after VSV infection was established resulted also in a drastic reduction of viral replication, as demonstrated by the dose-dependent decrease in the percentage of infected cells and GFP intensity (**Figure 2F**). This last observation corroborates the potential of VLPs-M8 as antiviral agent, extending its activity also to a therapeutic application.

### Antiviral activity elicited by VLPs-M8 is strictly dependent by the RIG-I – IFN axis

**Figure 3:**
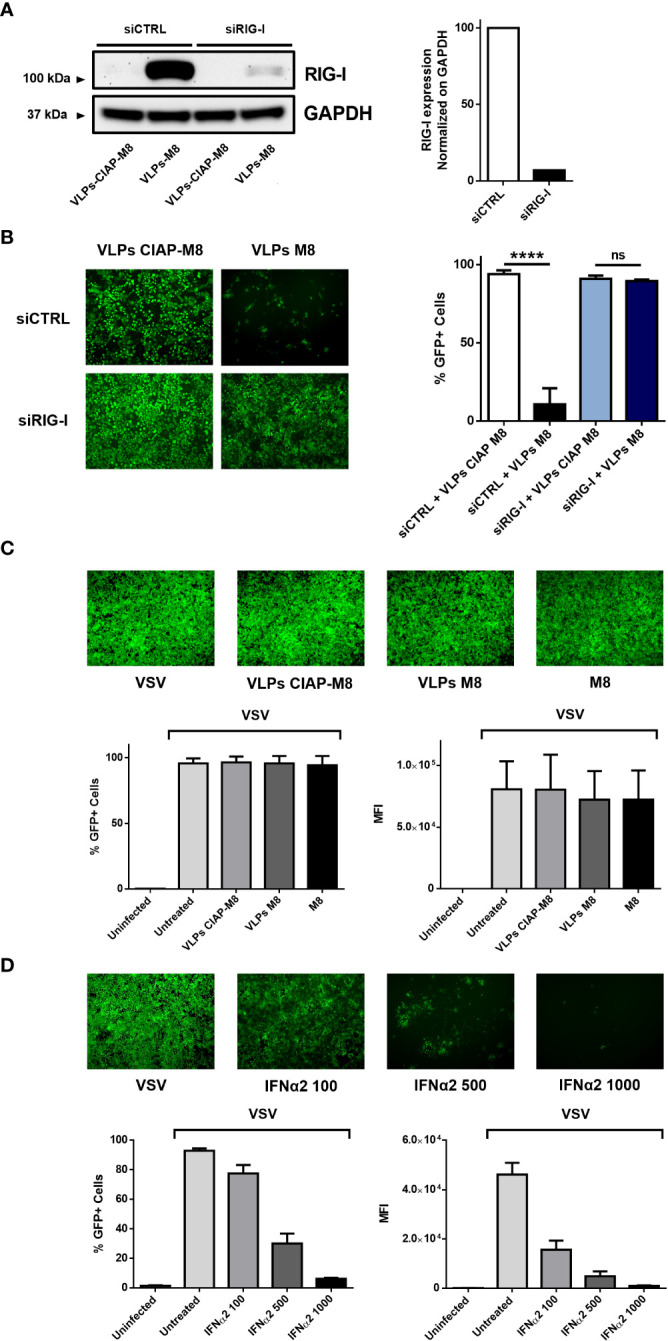
VLPs-M8 activity relies on RIG-I and type-I IFN signaling. **(A)** RIG-I expression in A549 cells. A549 cells were transfected with 10pmol of scrambled siRNA (siCTRL) or siRIG-I for 72h and immunoblot analysis of RIG-I expression was performed on whole cell extract; GAPDH was used as endogenous control for normalization. On the right, schematic representation of RIG-I protein expression, normalized on GAPDH and indicated as folds over control (siCTRL) set as 100. Image Lab software was used to quantify the protein bands and ratio was calculated by using software volume tool comparing lanes 2 and 4 (Bio-Rad, Hercules, CA, USA). **(B)** VSV-GFP infection in silenced A549 cells. Left, fluorescence microscopy images representative of A549 cells silenced with a scrambled siRNA (siCTRL) or siRIG-I for 24h, then treated with the indicated VLPs for 48h and infected with VSV-GFP (MOI 1) for further 24h. On the right, flow cytometry analysis indicating the percentage of GFP_+_ silenced A549 cells treated with VLPs and infected for 24 with VSV-GFP (MOI 1). **(C)** VSV-GFP infection in VERO E6 cells. Upper panel shows fluorescence microscopy images representative of VERO E6 treated with the indicated VLPs or M8 (10ng) directly transfected into cells and infected with VSV-GFP (MOI 0.1) for 24h. Bottom panels show flow cytometry analysis of percentage of GFP_+_ cells (left) and Mean Fluorescence Intensity (right). **(D)** VSV-GFP infection in VERO E6 cells treated with IFNα2. Upper panel shows fluorescence microscopy images representative of VERO E6 treated with increasing concentrations of IFNα2 (U/ml) for 24h and infected with VSV-GFP (MOI 0.1) for further 24h. Bottom panels show flow cytometry analysis of percentage of GFP_+_ cells (left) and Mean Fluorescence Intensity (right). Fluorescence images in **(B-D)** were obtained using a Olympus fluorescence microscope (10x magnification) and analyzed by ImageJ. Data represent mean + SEM from 3 independent experiments. Statistical significance was defined as follows: ****p < 0.0001, ns, not significant.

The induction of type I IFN signaling in presence of M8 occurs upon its binding to RIG-I and the consequent activation of the downstream pathway. To evaluate whether VLPs-M8 stimulation of IFN production and activation of the antiviral response relied on RIG-I signaling, A549 cells were silenced for RIG-I gene expression (**Figure 3A**), then treated with VLPs-M8 or VLPs-CIAP-M8 and infected with VSV. The evaluation of GFP expression, performed by fluorescence microscopy and flow cytometry analysis at 24hpi showed that a >80% decrease in VSV infection was detected with VLPs-M8 in presence of the scrambled siRNA negative control, while when RIG-I was silenced, this reduction was abrogated, and no statistically significative differences were observed (**Figure 3B**). To further confirm the role of type I IFN in VLPs-M8 – induced antiviral response, we treated and infected VERO E6 cells, an African Green Monkey kidney cell line defective in type I IFNs production but sensitive to exogenous IFNs (Emeny and Morgan, 1979). As expected, VERO E6 cells were unable to block VSV infection either upon VLPs-M8 treatment or with direct transfection of M8 (**Figure 3C**); on the other hand, treatment with increasing doses of IFN-α2a progressively decreased the number of infected cells (**Figure 3D**). These data confirm the dependence of VLPs-M8 – induced antiviral response on the RIG-I – mediated type I IFN production, further highlighting the specificity of molecular signaling triggered by this formulation.

### VLPs-M8 protect cells against DENV, VSV-Spike and hCoV-229E infections

**Figure 4:**
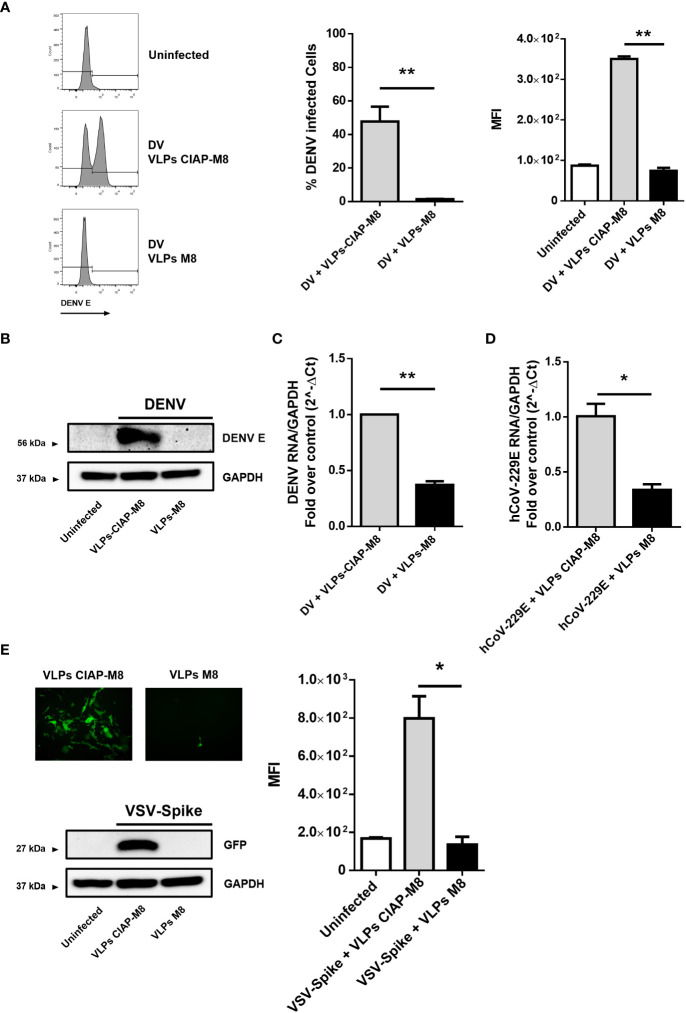
VLPs-M8 target different cells and restrict a broad range of viral infections **(A)** Analysis of DENV infection in A549 cells. Left, representative histograms of DENV E protein expression in infected A549 cells in presence of VLPs-CIAP-M8 or VLPs-M8 determined by flow cytometry using a mouse IgG2a mAb, specific for DENV E protein (clone 4G2); central and right panels show percentage of GFP_+_ cells and Mean Fluorescence Intensity, respectively. **(B)** Immunoblot analysis representative of DENV E protein expression in A549 whole cell extract. Cells were treated with VLPs as indicated for 48h and infected with DENV (MOI 2) for 24h; protein expression was normalized on GAPDH. **(C)** DENV NS4A RNA expression levels were evaluated by qPCR analysis in A549 cells after 24h of infection. Gene expression was normalized to GAPDH expression and is presented as RNA levels relative to those in VLPs-CIAP-M8 treated cells, set by default at 1. **(D)** hCoV-229E RNA expression levels were evaluated by qPCR analysis in Huh-7 cells after 24h of infection. Gene expression was normalized to GAPDH expression and is presented as RNA levels relative to VLPs-CIAP-M8 treated cells, set by default at 1. **(E)** Fluorescence microscopy images obtained using a Olympus fluorescence microscope (10x magnification) representative of Calu-3 cells treated with VLPs-CIAP-M8 or VLPs-M8 and infected with Spike-VSV-GFP (MOI 0.1) for 72h. Bottom panel shows the immunoblot analysis representative of GFP protein expression in Calu-3 whole cell extract. Cells were treated with VLPs as indicated for 48h and infected with Spike-VSV-GFP (MOI 0.1) for 72h; protein expression was normalized on GAPDH. Right panel, Mean Fluorescence Intensity (MFI) of GFP measured by flow cytometry. Data represent mean + SEM from 3 independent experiments. Statistical significance was defined as follows: *p < 0.05, **p < 0.01.

To determine the magnitude of antiviral response and the range of activity of VLPs-M8, we next evaluated the ability of VLPs-M8 to block DENV infection in A549 cells. Similar to what observed with VSV, treatment of A549 with VLPs-M8 dramatically inhibited DENV infection, as demonstrated by the complete absence of DENV E protein expression, evaluated either by flow cytometry (**Figure 4A**) or immunoblot analysis (**Figure 4B**), as well as a >60% reduction in viral RNA levels (**Figure 4C**).

To further assess the antiviral potential of VLPs-M8, we treated and infected hepatocellular carcinoma Huh-7 and lung carcinoma Calu-3 cells with the SARS-CoV-2 surrogate hCoV-229E and a VSV engineered to express the SARS-CoV-2 Spike and GFP (VSV-Spike), respectively. A reduction of ~70% in viral RNA levels was achieved in Huh-7 cells infected with hCoV-229E in the presence of VLPs-M8, compared to VLPs-CIAP-M8 (**Figure 4D**). Finally, a complete suppression of VSV-Spike infection was achieved in Calu-3 cells, as highlighted by fluorescence microscopy, flow cytometry measurement and immunoblot analysis of GFP expression (**Figure 4E**). These results further support the protective potential of VLPs-M8, demonstrating the ability to target different cell types and generate a strong antiviral immune response.

### Spike-VLPs-M8 selectively targets ACE2_+_ cells

**Figure 5:**
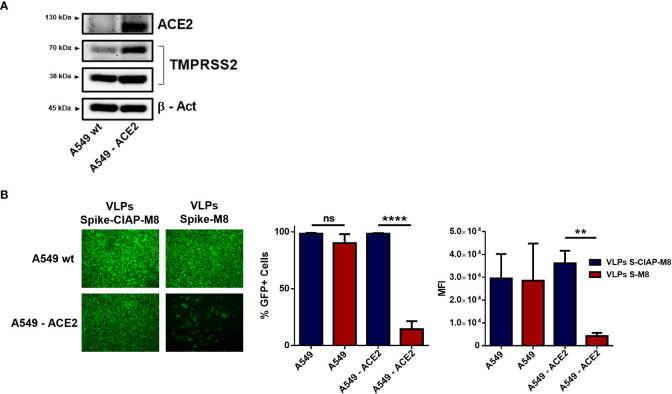
Expression of SARS-CoV-2 spike retargets VLPs to ACE2_+_ cells. **(A)** Immunoblot analysis representative of ACE2 and TMPRSS2 protein expression in A549 vs A549-ACE2 whole cell extracts, β-Actin was used for normalization. **(B)** VSV-GFP infection in A549 vs A549-ACE2 cells. Left panel shows fluorescence microscopy images representative of A549 and A549-ACE2 cells treated with Spike-VLPs-CIAP-M8 or Spike-VLPs-M8 and infected with VSV-GFP (MOI 0.01) for 24h; images were obtained using a Olympus fluorescence microscope (10x magnification) and analyzed by ImageJ. Histograms show flow cytometry analysis indicating the percentage of GFP_+_ cells and the Mean Fluorescence Intensity in A549 and A549-ACE2 cells, treated with Spike-VLPs-CIAP-M8 (blue bars) or Spike-VLPs-M8 (red bars). Data represent mean + SEM from 3 independent experiments. Statistical significance was defined as follows: **p < 0.01 and ****p < 0.0001, ns, not significant.

The possibility to express different surface molecules makes VLPs a versatile platform for cell-specific targeting. To verify whether VLPs engineered to express SARS-CoV-2 Spike surface glycoprotein (VLPs-S-M8) were able to selectively bind to ACE2 expressing (ACE2_+_) cells, we generated a stable cell line by transducing A549 cells with a lentiviral vector expressing ACE2 receptor (A549-ACE2) (**Figure 5A**) and evaluated the ability of VLPs-S-M8 to elicit an antiviral response in A549-wt and A549-ACE2 cells infected with VSV-GFP. As expected, the virus was able to infect both wild type and ACE2_+_ cells: however, only ACE2_+_ cells were targeted by VLPs-S-M8 which triggered an antiviral response that resulted in a significant reduction of viral replication (>80%), as compared to wild type or VLPs-S-CIAP-M8 treated cells (**Figure 5B**). These observations demonstrate the adaptability of VLPs as a delivery platform, highlighting the specificity towards a selective target and further emphasizing their therapeutical potential in settings where a non-systemic response is required.

## Discussion

The use of nanoparticles (NPs) for vaccination has become a promising strategy in recent years and several commercially available vaccines already employ this technology. The advantages of using NPs lie in their size, usually <1000 nm, the possibility to express various antigens on particles surface and the opportunity to shield in their inner core drugs, proteins, nucleic acids and immunostimulatory molecules for targeted delivery. The rapid development of SARS-CoV-2 vaccines is a clear demonstration of the extraordinary efforts made by the scientific community to combat the COVID-19 pandemic, with the release of several approved vaccines and other candidates currently undergoing clinical trials (Alexandridi et al., 2022). Among them, six candidates are based on Virus-Like Particles (VLPs) platform (WHO, 2022). VLPs represent the most promising NPs for vaccine production against infectious diseases, although several studies are also exploring their use as a potent therapeutic system to treat cancers, chronic inflammatory diseases, or neurodegenerative disorders such as Alzheimer’s disease and Parkinson’s disease (Mohsen and Bachmann, 2022). The expression of immunostimulatory antigens in a repetitive pattern on VLPs surface and the possibility to build empty VLPs scaffolds and subsequently link antigens by chemical or genetic fusion techniques, substantially increased their immunogenicity compared to traditional or subunit vaccines (Mohsen et al., 2017). Upon VLPs detection by APCs, the processed antigens are then loaded onto MHC-II molecules and cross-presented by MHC-I. As a consequence, these events result in the priming of both CD4_+_ and CD8_+_ T-cells, leading to a strong stimulation of antigen-specific acquired immune response and the generation of robust correlates of protection (Moffat et al., 2013; Mohsen et al., 2017; Yang et al., 2017; Serradell et al., 2019). The inherent capacity to trigger an immune response resides in the molecular composition of VLPs, which is similar to a native virus and can be further potentiated by the concomitant administration of adjuvants including Pattern Recognition Receptor (PRR) activators. Agonists already included in licensed vaccines (Didierlaurent et al., 2009; Cimica and Galarza, 2017; Mohsen et al., 2017; Pillet et al., 2022), represent potent activators of both innate and adaptive immunity and their application as adjuvants into VLPs is attracting significant interest (Thaiss et al., 2016; Pulendran et al., 2021).

The cytosolic sensor of short viral dsRNA, RIG-I, represents an ideal target for VLP-adjuvant formulations, since RIG-I agonists possess strong antiviral activity and induce cellular and humoral immune response (Suthar et al., 2010; Kandasamy et al., 2016; Yong and Luo, 2018; Chen et al., 2019; Heidegger et al., 2019a; Heidegger et al., 2019b). In the present work, we generated VLPs incorporating M8, a 5’ppp-RNA that activates type I IFNs signaling in a RIG-I – specific and MDA5-TLR3 – independent manner and evaluated the ability of VLPs-M8 to deliver the 5’ppp-RNA to different types of cells *in vitro*, with the consequent induction of IFN-I response and inhibition of viral replication. Previous studies have demonstrated the strong antagonism exerted by M8 against viruses and cancer (Olagnier et al., 2014; Beljanski et al., 2015; Chiang et al., 2015; Castiello et al., 2019). Furthermore, Beljanski et al. showed that M8 possesses strong adjuvant proprieties when used alongside VLPs-based influenza vaccines: co-treatment of mice with M8 and VLPs expressing neuraminidase (NA) and hemagglutinin (HA) from H5N1 influenza virus, protected the animals from lethal doses of influenza virus and induced a long-term antibody response compared to the combination of VLPs and other adjuvants (Beljanski et al., 2015). On the other hand, the incorporation of M8 into VLPs and its antiviral and immunogenic potential within this formulation has never been tested. Here, we report that VLPs built upon a HIV-1 packaging core and decorated with VSV-G glycoprotein, spontaneously assembled in HEK293T cells and incorporated M8, as demonstrated by the fluorescence expression detected in cells transduced with VLPs carrying a modified M8 conjugated with Cy-3 fluorophore. VLPs efficiently released M8 in A549 lung adenocarcinoma cells and potently induced transcription of IFN-β and ISGs, with only a weak induction of pro-inflammatory cytokines. This phenomenon could represent a critical advantage in settings that require the generation of a strong antiviral response without activating a potentially detrimental inflammatory reaction. When A549 were infected with VSV, a 48h pre-treatment with VLPs-M8 conferred to these cells an antiviral state that dramatically reduced the levels of infection (≈<90%) as compared to A549 transduced with VLPs incorporating the inactive form of M8 (CIAP-M8) or with VLPs defective of packaging (MD2.G-M8) or envelope (PAX2-M8). The antiviral activity observed in these cells relied exclusively on the RIG-I – IFN axis triggered by M8, as demonstrated by the abrogation of M8 protective effect against VSV infection upon RIG-I silencing. Furthermore, VERO E6 cells - that lack type I IFN production - were insensitive to the administration of VLPs-M8 or M8 alone and were consequently unable to control VSV infection. Conversely, addition of exogenous hIFNα2a reduced viral levels in a dose – dependent manner.

VLPs-M8 possessed not only a prophylactic potential but also a therapeutic capacity: when administered to A549 cells after the onset of VSV infection, M8 strongly suppressed viral replication as was observed in pre-treated conditions. This result suggests that VLPs-M8 may represent a therapeutic treatment for acute viral infections and corroborates previous studies demonstrating the ability of M8 to inhibit ongoing dengue and chikungunya virus replication *in vitro* and *in vivo*, respectively (Olagnier et al., 2014; Chiang et al., 2015). In addition, M8 blocked the infection of drug-resistant influenza virus strains, thereby indicating a stronger efficacy compared to direct antiviral drugs (Chiang et al., 2015). Further studies, including *in vivo* models of infection, are required to investigate the therapeutic potential of VLPs-M8 and to fully characterize the effects of post-infection application. Moreover, the inclusion into VLPs represents a powerful system for M8 delivery to different cell types. Indeed, VLPs also released M8 in Calu-3 and Huh-7 cells and the antiviral activity elicited by M8 blocked DENV, VSV-Spike and hCoV-229E virus replication.

The ability to direct VLPs to multiple cells is provided by the broad tropism of the VSV-G protein expressed on the particle surface, which recognizes the highly ubiquitous low density lipoprotein receptor (LDLR) (Finkelshtein et al., 2013). However, the natural tropism of VLPs represents a drawback when the targeting of other sites is desired. Herein, we demonstrated that incorporation of the SARS-CoV-2 Spike glycoprotein on VLPs surface retargeted these particles towards ACE2 expressing cells, and blocked VSV infection in A549-ACE2 but not in wild type cells. Altogether, the expression of an alternative cell-specific ligand can retarget VLPs to a distinct receptor and therefore potentially deliver therapeutic cargoes to specific tissues or organs.

These observations support the growing body of studies that identifies VLPs as a intriguing system for targeted therapies: several models have been developed not only in vaccination strategies, where the expression of selective antigen domains elicit the production of neutralizing antibodies (Zha et al., 2021), but also for the delivery of therapeutic molecules by expressing ligands for specific receptors overexpressed in tumor cells (Pokorski et al., 2011; Kitai et al., 2011; Galaway and Stockley, 2012; Liu et al., 2022) or for gene editing applications (Banskota et al., 2022). Overall, in this study we have demonstrated several key points that make the VLPs-M8 system a promising platform for a potential clinical use: *I) Inclusion –* we demonstrated that HIV-1 - based packaging system incorporated and protected M8 for intracellular release; *II) Delivery* M8 was efficiently transferred to target cells where it triggered a RIG-I – dependent type-I IFN response that drastically reduced or completely blocked viral replication; *III) Versatility and Specificity –* one of the most important advantages of this system is its flexibility; indeed VLPs surface modifications guide VLPs to a selective cell population expressing a specific receptor. Based on the present studies, we suggest a potential therapeutic application in the context of SARS-CoV-2 infection. VLP-mediated delivery to ACE2-expressing permissive cells could generate a robust antiviral response that could mitigate SARS-CoV-2 infection while avoiding an exacerbated inflammatory reaction or cytokine storm leading the development of severe COVID-19 (Hsu et al., 2022). Further improvements in this platform, including modifications for increased delivery and immunogenicity, will be required for *in vivo* applications.

## Methods

### Cell lines and plasmids

HEK293T, Huh-7 and Vero E6 cells were maintained in Dulbecco’s modified Eagle’s medium (DMEM) in presence of heat-inactivated 10% fetal bovine serum (FBS; Gibco), 300 ug/mL L-glutamine (Life Technologies), 100 U/mL penicillin and 100 μg/mL streptomycin (Sigma-Aldrich). A549 cells were grown in Ham’s F-12K (Kaighn’s) Medium (Thermo Fisher Scientific) supplemented with heat-inactivated 10% FBS (Gibco), 100 U/mL penicillin and 100 μg/mL streptomycin (Sigma-Aldrich). Calu-3 epithelial lung cancer cells (kindly provided by Prof. David Olagnier, Aarhus University, Aarhus C 8000, Denmark) were grown in MEM alpha (Euroclone), supplemented with heat-inactivated 20% FBS (Gibco), 300 ug/mL L-glutamine (Life Technologies), 100 U/mL penicillin and 100 μg/mL streptomycin (Sigma-Aldrich). A549-ACE2 cells were generated by transduction of A549 cells with lentivirus expressing ACE2-TMPRSS2 (Addgene #154987) and selection with puromycin 1 μg/mL. pWPI-IRES-Puro-Ak-ACE2-TMPRSS2 was a gift from Sonja Best (Addgene plasmid # 154987; http://n2t.net/addgene:154987; RRID : Addgene_154987); pMD2.G (Addgene plasmid # 12259; http://n2t.net/addgene:12259; RRID : Addgene_12259) and psPAX2 (Addgene plasmid # 12260; http://n2t.net/addgene:12260; RRID : Addgene_12260) were a gift from Didier Trono; CoV2-Spike-D614G (Addgene plasmid # 177960; http://n2t.net/addgene:177960; RRID : Addgene_177960), CoV2-M-IRES-E (Addgene plasmid # 177938; http://n2t.net/addgene:177938; RRID : Addgene_177938) and CoV2-N-S202R (Addgene plasmid # 177950; http://n2t.net/addgene:177950; RRID : Addgene_177950) were a gift from Jennifer Doudna.

### M8 generation

M8 was synthesized using Megascript T7 Transcription Kit (Thermo Fisher Scientific) with synthetic oligonucleotides (Eurofins Genomics) and following manufacturer instructions. Templates used were:

Fw: GAA ATT AAT ACG ACT CAC TAT AGA CGA AGA CCA CAA AAC CAG ATA AAA AAA AAA AAA AAA AAA AAAAAA ATA ATT TTT TTT TTT TTT TTT TTT TTT TTT ATCTGG TTT TGT GGT CTT CGT C

Rev: GAC GAA GAC CAC AAA ACC AGA TAA AAA AAA AAA AAA AAA AAA AAA AAA TTA TTT TTT TTT TTT TTT TTT TTT TTT TTA TCT GGT TTT GTG GTC TTC GTC TAT AGT GAG TCG TAT TAA TTT C

Synthesized RNA was then purified using Nucleospin MiRNA Kit (Macherey-Nagel) and its concentration was assessed using Nanodrop 2000 (Thermo Fisher Scientific).

To remove the 5′ triphosphate group of M8 (CIAP-M8), Calf Intestinal Alkaline Phosphatase (Thermo Fischer Scientific) was used following manufacturer instructions and RNA was then purified as above.

### M8 labeling

M8 – cyanine-3 was generated using the Label IT^®^ Nucleic Acid Labeling Kit (Mirus Bio LLC) following manufacturer instructions. Briefly, M8 was mixed with Labeling Reagent and Buffer and incubated at 37°C for 1h. After incubation, labeled M8 was purified by ethanol precipitation: 0.1 volume of 5M sodium chloride and 2.5 volumes of ice cold 100% ethanol were added to the reaction; the mix was stored at -20°C for 30 minutes and then centrifuged at 17,000 x g for 30 minutes. Pellet was then washed with 500 μl room temperature 70% ethanol and centrifuged at 17,000 x g for 30 minutes. Finally, labeled M8 was resuspended in molecular biology-grade water and concentration was measured with Nanodrop 2000 (Thermo Fisher Scientific).

### VLPs generation and quantification

VLPs were generated by transient transfection of HEK293T cells with polyethylenimine (PEI) at a concentration of 14 µg/µg of DNA or RNA. HEK293T were seeded in a T-75 flask and transfected at 70% confluency; VLPs were produced by co-transfection of plasmids encoding VSV-G envelope (pMD2.G, Addgene #12259) and packaging (psPAX2, Addgene #12260) at a ratio of 1:2, and 1 µg of M8 or CIAP-M8 (control VLPs). Medium was changed 24h after transfection and replaced by fresh complete DMEM. 48h after transfection supernatant was collected, centrifuged at 300 x g for 5 minutes and filtered with a 0.45 µm pore size hydrophilic polyethersulfone (PES) membrane. VLPs were then purified and concentrated by ultracentrifugation on a 20% glycerol cushion at 135,000 x g for 4h at 4°C using a Beckman SW32Ti Swing Bucket Rotor. Concentrated VLPs were resuspended in PBS. Spike-VLPs were generated as previously described (Syed et al., 2021); briefly, HEK293T cells were seeded in a T-75 flask and transfected at 70% confluency; VLPs were produced by co-transfection of plasmids encoding SARS-CoV-2 Spike (Addgene #177960), M-E (Addgene #177938) and N proteins (Addgene #177950) at a ratio of 0.0016:0.33:0.67 for a total of 4 μg of DNA and 1 µg of M8 or CIAP-M8. Spike-VLPs purification and concentration was performed as described above. An amount of 20 μg and 40 μg was used in this study for VLPs and Spike-VLPs, respectively. VLPs-Cy3 titer was determined by flow cytometry analysis of Cyanine-3 expression in Vero E6 cells (%Cy-3 positive cells*#infected cells/mL of VLPs). Titers were expressed as IU/ml. For cells transduction, VLPs were diluted in complete medium at the moment of treatment and left for 48 hours, until viral infection was performed in serum free medium; after 1h of incubation with the virus, VLPs were then removed and medium was replaced with complete fresh medium.

### Virus production, quantification, and infection

wtVSV-GFP (Indiana serotype) was propagated in VERO E6 cells; briefly, VERO E6 were infected with VSV-wt at MOI of 0.01 for 48h, supernatant was collected, centrifuged 5’ at 300 x g and then filtered using a 0.22µm bottle-top vacuum filter. Virus was concentrated by ultracentrifugation at 18,000 x g for 90’ at 4°C and then purified on a 20% sucrose cushion at 135,000 x g for 90’ at 4°C using a Beckman SW32Ti Swing Bucket Rotor. Purified virus was resuspended in PBS and virus titer was quantified by a standard plaque assay method on BHK-21T7 cells as described previously (Nguyên et al., 2008).

Dengue virus production was performed as previously described (Ferrari et al., 2020). Briefly, C6/36 cells were infected with DENV-2 NGC at low MOI (0.05); after 7 days supernatant of infected cells was collected and cleared by centrifugation. The virus was then concentrated and purified by ultracentrifugation on a 20% sucrose cushion. In infection experiments, A549 cells were infected in serum-free medium for 1h at 37°C and then incubated with complete medium for 24h prior to analysis. Viral infection and titer were determined by flow cytometry analysis of DENV E protein expression in A549 and Vero E6 infected cells, respectively (Lambeth et al., 2005). Titers were expressed as IU/ml. hCoV-229E (ATCC VR-740) was grown and maintained in Huh-7 cells. VSV-Spike-GFP is a replication-competent VSV expressing eGFP in the first position of the genome as well as a modified version of the SARS-CoV-2 spike in place of the native VSV glycoprotein, was a kind gift of Prof. David Olagnier (Aarhus University, Aarhus C 8000, Denmark) and was obtained from Prof. Paul W. Rothlauf as described (Case et al., 2020).

### Quantitative PCR

RNA was isolated by column separation using the RNeasy Kit (Qiagen) following manufacturer’s instructions and the concentration was measured with Nanodrop 2000 (Thermo Fisher Scientific). A quantity of RNA in the range 200 – 500 ng was used for cDNA synthesis using the PrimeScript RT-PCR Kit (Takara-Bio). Quantitative PCR was then performed using Taqman Fast Advanced MasterMix with Universal Probe Library Probes (Roche) with specific primers designed using the Roche Lifescience Assay Design Center (https://lifescience.roche.com/en_it/brands/universal-probe-library.html#assay-design-center) on a StepOnePlus Real-Time PCR System (Thermo Fischer Scientific). A relative quantification method was used, with GAPDH or Beta-Actin as housekeeping genes. Primers used in this study: IFN-β Fw-CTTTGCTATTTTCAGACAAGATTCA, Rev-GCCAGGAGGTTCTCAACAAT; ISG15 Fw-GCGAACTCATCTTTGCCAGTA, Rev-CCAGCATCTTCACCGTCAG; ISG56 Fw-GCCTAATTTACAGCAACCATG, Rev-TCA TCAATGGATAACTCCCATGT; CXCL10 Fw-GAAAGCAGTTAGCAAGGAAAG, Rev-GACATATACTCCATGTAGGGAAGTGA; IL-6 Fw-GATGAGTACAAAAGTCCTGATCCA, Rev-CTGCAGCCACTGGTTCTGT; TNF-α Fw-GACAAGCCTGTAGCCCATGT, Rev-TCTCAGCTCCACGCCATT; DENV NS4A Fw-ATCCTCCTATGGTACGCACAAA, Rev-CTCCAGTATTATTGAAGCTGCTATCC; hCoV-229E Fw-ACCAACATTGGCATAAACAG, Rev-CGTTGACTTCAAACCTCAGA; β-Actin Fw-ATTGGCAATGAGCGGTTC, Rev-TGAAGGTAGTTTCGTGGATGC; GAPDH Fw-AGCCACATCGCTCAGACA, Rev-GCCCAATACGACCAAATCC.

### Protein extraction and immunoblot analysis

Cells were washed in ice-cold PBS and lysed in lysis buffer (150 mM NaCl, 50 mM Tris-HCl, pH 7.6, 5mM EDTA pH 8, 1% Nonidet -P-40 (NP-40), 0,5% sodium deoxycholate, 0.1% sodium dodecyl sulfate (SDS)), in the presence of Halt™ Protease and Phosphatase Inhibitor Cocktail (Thermo Fisher Scientific), and benzonase (Sigma-Aldrich) for 25 min on ice. Protein concentration was determined using the Pierce bicinchoninic (BCA) protein assay kit (Thermo Scientific). Proteins were resolved by SDS-PAGE on 4%–20% precast Novex Tris-Glycine gradient gels (Thermo Fisher Scientific) and blotted onto polyvinylidene difluoride (PVDF) membranes (GE Healthcare). Blots were incubated with the indicated primary antibodies, at 1:1000 dilution in 5% (w/v) BSA, overnight at 4°C, extensively washed with TBS-T and after incubation with horseradish peroxidase (HRP)-labelled goat anti-rabbit or goat anti-mouse Abs (Cell Signaling Technology), developed with the enhanced chemiluminescence (ECL) detection system as per manufacturer’s instructions (Cyanagen). Primary antibodies anti-phospho-IRF3 Ser396 (#4947), anti-IRF3 (#11904), anti-RIG-I (#3743), anti-ACE2 (#15983) and anti-*β*-actin (#4967) were all purchased from Cell Signaling Technology (Danvers, Massachusetts, USA). Primary antibodies anti-GAPDH (sc-47724), anti-DENV-E (sc-325014), anti-GFP (sc-9996), anti-HIV-p24 (sc-69728), anti-TMPRSS2 (sc-515727) and anti-VSV-G (sc-66180) were all purchased from Santa Cruz Biotechnology (Dallas, TX, USA). Secondary antibodies anti-Mouse IgG, HRP-linked Antibody (#7076) and Anti-rabbit IgG, HRP-linked Antibody (#7074) were purchased from Cell Signaling Technology (Danvers, Massachusetts, USA).

### Fluorescence microscopy

Images of Cyanine3- and GFP- positive cells were obtained using an Olympus fluorescence microscope. For nuclear staining, samples were mounted with ProLong Diamond Antifade Mountant with DAPI (Cat. #P36966, Invitrogen/ThermoFisher Scientific, Waltham, MA, USA).

### Flow cytometry

All the experiments were performed on BD FacsCanto II (BD Biosciences). For wtVSV-GFP, VSV-Spike-GFP infection and for detection of M8-Cy3 expression, cells were harvested at the indicated time, washed twice in 1x PBS and finally resuspended in 100µl 1x PBS prior to FACS analysis. For DENV infection and titer measurement, infected cells were harvest 24hpi and fixed with 4% paraformaldehyde for 10’ at room temperature (RT); after incubation, cells were washed twice in 1x PBS and permeabilized with Permeabilization Buffer (0.25% Saponin + 2% FBS, in PBS 1x) for 15’ at RT. Cells were then washed twice in permeabilization buffer and incubate with anti-DENV E antibody for 30’ at RT, then washed again in buffer and incubated for another 30’ with PE-anti-mouse secondary antibody. Finally, cells were washed twice in permeabilization buffer, resuspended in 1x PBS and FACS analysis was performed to detect the percentage of DENV E positive cells (PE_+_).

## Data availability statement

The original contributions presented in the study are included in the article/supplementary material. Further inquiries can be directed to the corresponding author.

## Author contributions

EP contributed to the conception, experimentation and preparation of the manuscript and figures. MA, DC and MM participated in the experimentation and preparation of the manuscript, including text and figures. JH provided supervision, wrote and edited the manuscript. All authors contributed to the article and approved the submitted version.
